# Fear, anxiety and depression among pregnant women during COVID-19 pandemic: impacts of healthy eating behaviour and health literacy

**DOI:** 10.1080/07853890.2021.2001044

**Published:** 2021-11-11

**Authors:** Thuc C. Luong, Thu T. M. Pham, Minh H. Nguyen, Anh Q. Do, Linh V. Pham, Hoang C. Nguyen, Huu C. Nguyen, Tung H. Ha, Hung K. Dao, Manh V. Trinh, Thinh V. Do, Hung Q. Nguyen, Thao T. P. Nguyen, Cuong Q. Tran, Khanh V. Tran, Trang T. Duong, Hai X. Pham, Thao T. Do, Phuoc B. Nguyen, Anh L. Tra, Dung T. Phan, Binh N. Do, Tuyen Van Duong

**Affiliations:** a Department of Cardiology, Cardiovascular Center, Military Hospital 103, Hanoi, Vietnam; b Director Office, Military Hospital 103, Hanoi, Vietnam; c School of Public Health, College of Public Health, Taipei Medical University, Taipei, Taiwan; d Faculty of Public Health, Hai Phong University of Medicine and Pharmacy, Hai Phong, Vietnam; e International Ph.D. Program in Medicine, College of Medicine, Taipei Medical University, Taipei, Taiwan; f International Ph.D. Program for Cell Therapy and Regeneration Medicine, College of Medicine, Taipei Medical University, Taipei, Taiwan; g Department of Obstetrics and Gynecology, Hai Phong University of Medicine and Pharmacy, Hai Phong, Vietnam; h Department of Pulmonary and Cardiovascular Diseases, Hai Phong University of Medicine and Pharmacy Hospital, Hai Phong, Vietnam; i Director Office, Hai Phong University of Medicine and Pharmacy Hospital, Hai Phong, Vietnam; j Director Office, Thai Nguyen National Hospital, Thai Nguyen, Vietnam; k President Office, Thai Nguyen University of Medicine and Pharmacy, Thai Nguyen, Vietnam; l Director Office, E Hospital, Hanoi, Vietnam; m Department of Thoracic and Cardiovascular Surgery, E Hospital, Hanoi, Vietnam; n Director Office, General Hospital of Agricultural, Hanoi, Vietnam; o Director Office, Bac Ninh Obstetrics and Pediatrics Hospital, Bac Ninh, Vietnam; p Director Office, Quang Ninh General Hospital, Quang Ninh, Vietnam; q Director Office, Bai Chay Hospital, Quang Ninh, Vietnam; r Director Office, Quang Ninh Obstetrics and Pediatrics Hospital, Quang Ninh, Vietnam; s Health Management Training Institute, University of Medicine and Pharmacy, Hue University, Thua Thien Hue, Vietnam; t Director Office, Thu Duc District Health Center, Ho Chi Minh City, Vietnam; u Faculty of Health, Mekong University, Vinh Long, Vietnam; v Director Office, Le Van Thinh Hospital (previously Hospital District 2), Ho Chi Minh City, Vietnam; w Nursing Office, Tan Phu District Hospital, Ho Chi Minh City, Vietnam; x Department of Oral Pathology and Periodontology, Faculty of Odonto-Stomatology, Can Tho University of Medicine and Pharmacy, Can Tho, Vietnam; y Director Office, Kien An Hospital, Hai Phong, Vietnam; z Department of Rehabilitation & Physiotherapy, Da Nang University of Medical Technology and Pharmacy, Da Nang, Vietnam; aa Nursing Office, Thien An Obstetrics and Gynecology Hospital, Hanoi, Vietnam; ab Faculty of Nursing, Hanoi University of Business and Technology, Hanoi, Vietnam; ac Department of Infectious Diseases, Vietnam Military Medical University, Hanoi, Vietnam; ad Division of Military Science, Military Hospital 103, Hanoi, Vietnam; ae School of Nutrition and Health Sciences, Taipei Medical University, Taipei, Taiwan

**Keywords:** Fear, anxiety, depression, health literacy, healthy eating behaviour, COVID-19, pregnant women

## Abstract

**Introduction:**

The COVID-19 pandemic has been influencing people’s psychological health, especially in pregnant women. We aimed to examine associated factors of fear of COVID-19, anxiety and depression among pregnant women during the pandemic where the impacts of healthy eating behaviour (HES) and health literacy (HL) were emphasized.

**Methods:**

A cross-sectional study was conducted between 14 February 2020 and 31 May 2020 in 18 health centres and hospitals across Vietnam. Data of 518 pregnant women were analysed, including socio-demographics, pregnant-related factors, HES, HL, health-related behaviours, fear of COVID-19 scale (FCoV-19S), anxiety (using the generalized anxiety disorder (GAD-7)) and depression (using the patient health questionnaire with 9 items (PHQ-9)). Regression analysis was utilized to explore the associations.

**Results:**

Pregnant women with higher scores of HES and HL had lower likelihood of anxiety (odds ratio, OR, 0.79; 95% confidence interval (95%CI), 0.73, 0.87; *p* < .001; and OR, 0.94; 95%CI, 0.90, 0.99; *p* = .018) and depression (OR, 0.84; 95%CI, 0.78, 0.91; *p* < .001; and OR, 0.96; 95%CI, 0.91, 0.99; *p* = .044), respectively. Pregnant women being employed had a lower FCoV-19S score (regression coefficient, *B*, −1.46; 95%CI, −2.51, −0.40; *p* = .007). Besides, other significant predictors of anxiety were eating healthier during the pandemic, unchanged or more physical activity, elevated gestational age and smoking. Other significant predictors of depression were eating healthier during the pandemic, elevated gestational age and smoking.

**Conclusions:**

Among others, HES and HL had positive impacts on protecting pregnant women against anxiety and depression. Improving HES and HL should be addressed as a strategic approach to improve reproductive health during the pandemic.

**KEY MESSAGE**
- The COVID-19 pandemic influences antenatal mental disorders with the higher level as opposed to that before the pandemic.
- Healthy eating behaviour and better health literacy (HL) had critical roles in lowering prenatal anxiety and depression during the COVID-19 crisis.
- Strategic approaches for improving healthy eating and HL should be recommended for protecting pregnant women from mental health problems during the pandemic.

## Introduction

The COVID-19 pandemic has sparked panic and psychological health issues worldwide [1]. The frequent performance of preventive measures (e.g. hand washing, masking, social distancing and isolation) during the pandemic introduces the obsession to people, which increases the risk of psychological damage [2]. In addition, the pandemic induced daily life changes, and job and income losses, which increased the risk of anxiety and depression [3].

Pregnant women are more susceptible to any effect of the COVID-19 crisis that needs to call for actions to protect this population [4]. During the pandemic, pregnant women have constraints in accessing essential healthcare services [5]; concerns over COVID-19 exposure, childcare, breastfeeding and vaccination [6], which further affect their psychological health. Although psychological alteration is one of the major characteristics during pregnancy, the occurrence and level of mental illness in pregnant women were much higher in the period of COVID-19 pandemic than the pre-pandemic period [7,8]. Fear of COVID-19, anxiety and depression were the most prevalent mental disorders among pregnant women [9,10]. Such disorders were associated with adverse pregnancy outcomes, such as preterm birth, small for gestational age and low infant birth weight [11,12].

Reports on the factors associated with mental disorders in pregnant women were available in the literature review, such as the lack of family support, marital conflict, history of obstetric complication, elevated gestational age, low income, food insecurity and young age [13,14]. During the COVID-19 pandemic, several valuable related factors were investigated. For example, the food insecurity caused by multilevel factors resulted in psychological stress through behaviour changes such as reducing fruit and vegetable consumption [15,16]. Paskulin et al. [17] reported that unhealthy dietary pattern (e.g. low fruits and bean intake, high sweets and sugars intake) was associated with high proportions of anxiety and depression. In addition, our previous studies found that healthy eating behaviour (HES) and health literacy (HL) impacted fear of COVID-19, anxiety and depression among and healthcare workers (HCWs) and general outpatients [18,19].

Although determinants of psychological health problems in pregnant women were reported, the number of studies investigating the effects of the COVID-19 crisis on antenatal mental health is limited. Therefore, we aimed to explore the associated factors of fear, anxiety and depression among pregnant women during the COVID-19 pandemic, where impacts of HES and HL were emphasized.

## Materials and methods

### Study design and population

**Figure 1.**
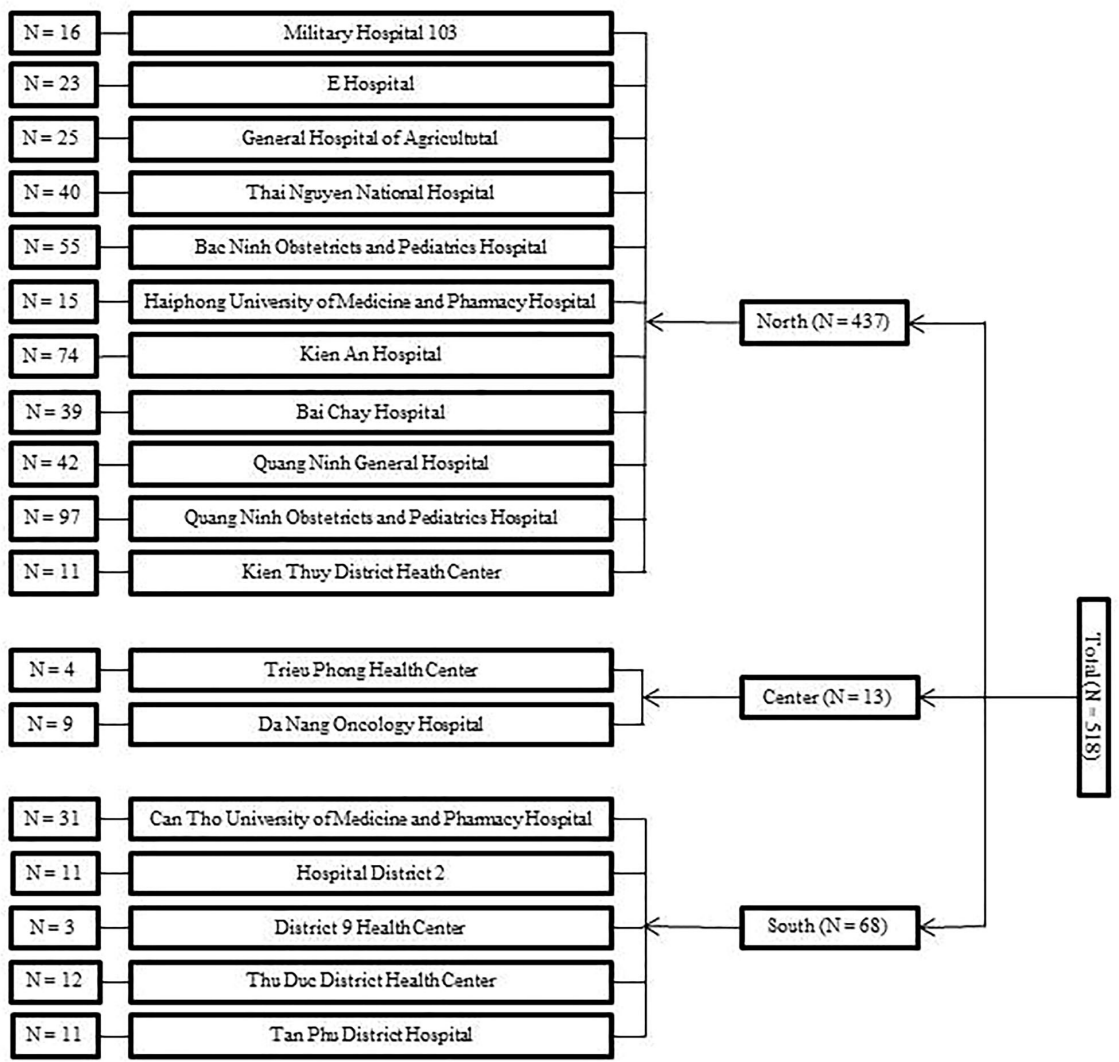
The study sample in hospitals and health centres across Vietnam.

A cross-sectional study was conducted from 14 February 2020 to 31 May 2020 in 18 health centres and hospitals across Vietnam. During COVID-19 pandemic, medical facilities spent their resources on controlling and treating patients with COVID-19. Therefore, we tried to engage hospitals as many as possible. Finally, 18 health centres and hospitals were available and agreed to participate in our study. Participants were those who visited the outpatient clinics and HCWs in the study settings. Outpatients were consecutively recruited, including those aged 18–85 years, without emergency conditions, and completed the survey. Out of 11,517 possible HCWs, we enrolled 7124 HCWs who aged 21–60 years and completed the survey. Besides, an estimated sample size of 424 was calculated using G-power software version 3.1.9.7 [20] with effect size of 0.05, type I error of 0.05, power of 0.95 and six predictors in the multiple linear regression. In our study, out of 8291 outpatients [19] and 7124 HCWs [18], a sample of 518 pregnant women were investigated which was larger than the calculated sample and satisfied for analysis. The participants were Vietnamese pregnant women who were not in an emergency condition, aged 18–40 years, and completed the self-administered questionnaires. The distribution of participants in the study settings is represented in Figure 1. During the study period, there were 328 confirmed COVID-19 cases and no death in Vietnam [21]. The infection prevention and control measures were applied in the procedure of data collection according to the guidelines of the Vietnam Ministry of Health [22] and World Health Organization [23], including using masks, washing hands and physical distance.

### Instruments and measurements

#### Demographic characteristics and clinical indicators

Pregnant women reported their age (years), occupation (employed, own business, others) and ability to pay for medication (very difficult to very easy). In addition, pregnancy-related data were self-report, including gestational age (weeks), the number of foetuses in the current pregnancy, gestational weight gain (kg) and parity. The gestational age was classified as the first, second and third trimester if the gestation was <17, between 17 and <29, and ≥29 weeks, respectively.

Participants also reported their suspected COVID-19 symptoms (S-COVID-19-S) [24], including fever, cough, dyspnoea, myalgia, fatigue, sputum production, confusion, headache, sore throat, rhinorrhoea, chest pain, haemoptysis, diarrhoea and nausea/vomiting. Participants were classified as having S-COVID-19-S if they had any of those symptoms. Finally, the comorbidity was assessed using the Charlson comorbidity index [25].

#### Health-related behaviours

The health-related behaviours within the COVID-19 period were reported as opposed to that before the pandemic, including eating behaviour (unchanged or less healthy vs. healthier), physical activity level (never, stopped, or less vs. unchanged or more). Since the proportions of smoking and drink in women were small, we regrouped and analysed the dichotomized variables of smoking (yes vs. no), and drinking (yes vs. no).

#### Healthy eating behaviour

Healthy eating behaviour was assessed using the 5-item healthy eating score (HES-5) to evaluate the overall diet quality. The HES-5 questionnaire was validated and used in previous studies [19,26,68]. Participants rated the frequency of eating or drinking fruits, vegetables, whole grains, dairy and fish in the previous 30 days based on the five-point Likert scale (e.g. 0 “rarely or never”, 1 “1–2 times/week”, 2 “3–6 times/week”, 3 “once/day”, 4 “twice/day”, 5 “≥3 times/day”). The total HES score ranged from 0 to 25, and a high score indicated good healthy dietary intake behaviour. Cronbach’s alpha of HES-5 was 0.77 in the current study.

#### Health literacy

Health literacy was assessed using a short-form survey with 12 items (HLS-SF12), which was validated and used widely in Vietnam [27,28]. The HLS-SF12 questionnaire was used to evaluate the ability to process health-related information in four stages, including accessing, understanding, appraising and applying [29]. In the current study, Cronbach’s alpha of HLS-SF12 was 0.93. Participants rated the difficulty levels of each item based on the four-point Likert scale from 1 (very difficult) to 4 (very easy). Then, the unified indices of HL from 0 to 50 were calculated with higher values representing better HL. The formula was mentioned in a previous study [30].

#### Assessment of outcome variables

The seven-item fear of COVID-19 scale (FCoV-19S) was used. This scale was validated and used in Vietnam [31,68,69]. Pregnant women responded to each item on a five-point Likert scale from 1 (strongly disagree) to 5 (strongly agree). Thus, the total scores ranged between 7 and 35, and a high score indicated a greater fear. In the present study, Cronbach’s alpha of FCoV-19S was 0.89.

Anxiety disorder was assessed using the Generalized Anxiety Disorder scale with seven items (GAD-7) [32] which was validated and used in Vietnam [33]. Pregnant women were asked about the frequency of having seven symptoms in the past two weeks on a four-point Likert scale, including 0=“not at all”, 1=“several days”, 2=“more than half the days” and 3=“nearly every day”. The total GAD-7 score ranged from 0 to 21, and score ≥8 represented pregnant women with anxiety [34]. In the current study, Cronbach’s alpha of GAD-7 was 0.93.

Depression was measured using the 9-item Patient Health Questionnaire (PHQ-9) [35], which was validated and used in Vietnam [36,37]. Pregnant women were asked about the frequency of being bothered with nine symptoms in the past two weeks and rated on the same four-point Likert scale as anxiety above. The total PHQ-9 score ranged from 0 to 27, and score ≥10 represented pregnant women with depression [38]. In the current study, Cronbach’s alpha of PHQ-9 was 0.90.

### Ethical consideration

This study was reviewed and approved by the Institutional Ethical Review Committee of Hanoi University of Public Health, Vietnam (IRB number: 029/2020/YTCC-HD3 and 133/2020/YTCC-HD3).

### Statistical analysis

First, we performed a descriptive analysis to explore the distribution of the different variables. We used chi-square tests (or Fisher’s exact test) for categorical variables and *t*-tests for continuous variables to compare the distribution of studied variables between women with and without anxiety or depression. In addition, we utilized *t*-tests (or one-way ANOVA test) to explore group differences in FCoV-19S. Second, the bivariate and multivariate linear regression models were used to analyse the associated factors of FCoV-19S. The bivariate and multivariate logistic regression models were used to analyse the associated factors of anxiety and depression. The factors associated with the outcome variables at *p* < .2 in the bivariate analyses were selected in the multivariate models. The correlation coefficients of <0.3 were tested using Spearman’s correlation for adjusted factors to avoid multicollinearity in the multivariate models (Supplementary Table 1). Regression coefficient (*B*), odds ratio (OR) and 95% confidence interval (95%CI) were reported, and a two-sided *p* value <.05 was considered statistically significant. All analyses were performed using IBM SPSS Version 22.0 (IBM Corp., Armonk, NY).

## Results

### Characteristics of study participants

As shown in Table 1, among the 518 pregnant women, the proportions of women with anxiety and depression were 8.1% and 8.7%, respectively. The mean FCoV-19S was 20.1 ± 5.3 and significantly different in the categories of occupation and physical activity. Pregnant women being employed and having an unchanged or more level of physical activity had a low mean of FCoV-19S with *p*=.001 and *p*=.023, respectively. Compared to participants without anxiety, those with anxiety had a higher gestational age (*p*=.010), a higher percentage of having negative lifestyle changes, including unchanged or less HES (*p* < .001), smoking (*p* < .001), drinking (*p*=.004) and never/stopped or exercise less (*p*=.005). Compared to participants without depression, those with depression had a higher gestational age (*p*=.022), a higher percentage of having negative lifestyle changes, including unchanged or less HES (*p* < .001), smoking (*p* < .001) and drinking (*p*=.001). In addition, pregnant women with anxiety and depression had a lower HES score than those without anxiety and depression (*p* < .001).

**Table 1.** Participants’ characteristics, fear of COVID-19, anxiety and depression.

Variables	Total (*n* = 518)	FCoV-19S (*n* = 514)		Anxiety (*n* = 513)			Depression (*n* = 513)		
		Mean ± SD	*p*	GAD < 8 (*n* = 476)	GAD ≥ 8 (*n* = 42)	*p*	PHQ < 10 (*n* = 473)	PHQ ≥ 10 (*n* = 45)	*p*
	*n* (%)			*n* (%)	*n* (%)		*n* (%)	*n* (%)	
Age (mean ± SD)	28.6 ± 4.0			28.6 ± 3.9	29.3 ± 5.1	.344	28.6 ± 3.9	28.9 ± 4.6	.648
Occupation			.001			.936			.204
Employed	385 (74.3)	19.6 ± 5.2		354 (74.4)	31 (73.8)		348 (73.6)	37 (82.2)	
Own business and others	133 (25.7)	21.3 ± 5.1		122 (25.6)	11 (26.2)		125 (26.4)	8 (17.8)	
Ability to pay for medication			.712			.705			.648
Very or fairly difficult	281 (54.5)	20.0 ± 5.0		257 (54.1)	24 (57.1)		258 (54.7)	23 (51.1)	
Very or fairly easy	235 (45.5)	20.2 ± 5.6		218 (45.9)	18 (42.9)		214 (45.3)	22 (48.9)	
S-COVID-19-S			.450			.152			.640
No	412(79.5)	19.9 ± 5.4		375 (78.8)	37 (88.1)		375 (79.3)	37 (82.2)	
Yes	106 (20.5)	20.4 ± 4.5		101 (21.2)	5 (11.9)		98 (20.7)	8 (17.8)	
Comorbidity			.257			.410			.426
None	496 (95.8)	20.1 ± 5.3		457 (96.0)	39 (92.9)		454 (96.0)	42 (93.3)	
One or more	22 (4.2)	18.8 ± 4.0		19 (4.0)	3 (7.1)		19 (4.0)	3 (6.7)	
Gestational age			.151			.010			.022
1st trimester	171 (33.3)	19.5 ± 4.5		167 (35.2)	5 (12.8)		166 (35.2)	6 (14.3)	
2nd trimester	136 (26.5)	19.8 ± 5.5		125 (26.3)	11 (28.2)		121 (25.6)	15 (35.7)	
3rd trimester	206 (40.2)	20.6 ± 5.6		183 (38.5)	23 (59.0)		185 (39.2)	21 (50.0)	
Gestational weight gain, median (IQR)	6.0 (2.0, 10.0)			6.0 (2.0, 11.0)	8.0 (5.0, 10.0)	.155	6.0 (2.0, 11.0)	9.0 (5.0, 10.0)	.155
Parity			.068			.904			.667
1	226 (43.6)	19.6 ± 5.0		208 (43.7)	18 (42.9)		205 (43.3)	21 (46.7)	
≥2	292 (56.4)	20.4 ± 5.5		268 (56.3)	24 (57.1)		268 (56.7)	24 (53.3)	
Number of foetuses			.100			.635			.663
1	500 (96.5)	20.1 ± 5.2		460 (96.6)	40 (95.2)		457 (96.6)	43 (95.6)	
≥2	18 (3.5)	18.0 ± 5.8		16 (3.4)	2 (4.8)		16 (3.4)	2 (4.4)	
Eating behaviour			.481			<.001			<.001
Unchanged or less-healthy diet	267 (51.5)	19.9 ± 4.6		232 (48.7)	35 (83.3)		230 (48.6)	37 (82.2)	
Healthier diet	251 (48.5)	20.2 ± 5.9		244 (51.3)	7 (16.7)		243 (51.4)	8 (17.8)	
Smoking			.587			<.001			<.001
No	483 (93.2)	20.0 ± 5.1		455 (95.6)	28 (66.7)		453 (95.8)	30 (66.7)	
Yes	35 (6.8)	20.7 ± 7.7		21 (4.4)	14 (33.3)		20 (4.2)	15 (33.3)	
Drinking alcohol			.942			.004			.001
No	429 (82.8)	20.1 ± 5.0		401 (84.3)	28 (66.7)		400 (84.6)	29 (64.4)	
Yes	89 (17.2)	20.1 ± 6.3		75 (15.7)	14 (33.3)		73 (15.4)	16 (35.6)	
Physical activity			.023			.005			.054
Never, stopped or exercise less	274 (52.9)	20.5 ± 5.0		243 (51.2)	31 (73.8)		244 (51.7)	30 (66.7)	
Unchanged or exercise more	244 (47.1)	19.5 ± 5.5		233 (48.8)	11 (26.2)		229 (48.3)	15 (33.3)	
HL index (mean ± SD)	33.6 ± 8.7			33.8 ± 8.5	31.4 ± 9.9	.086	33.8 ± 8.5	31.7 ± 10.2	.126
HES (mean ± SD)	14.3 ± 4.9			14.7 ± 4.6	9.2 ± 4.6	<.001	14.7 ± 4.7	10.0 ± 4.9	<.001
FCoV-19S (mean ± SD)	20.1 ± 5.3								

S-COVID-19-S: suspected COVID-19 symptoms; HL index: health literacy index; HES: healthy eating score; FCoV-19S: fear of COVID-19 scale.

### Associated factors of fear of COVID-19

In the bivariate models (Table 2), occupation, gestational age, parity, physical activity and HES were associated with FCoV-19S at *p* < .20. Additionally, there was no multicollinearity among those confounders (Supplementary Table 1). The multivariable linear regression analysis results in Table 2 indicated that employed pregnant women had a lower FCoV-19S score (regression coefficient, *B*= −1.46, 95%CI, −2.51, −0.40, *p*=.007) compared to those with other types of occupation.

**Table 2.** Associated factors of FCoV-19S in pregnant women.

Variables	FCoV-19S			
	Bivariable		Multivariable	
	*B* (95%CI)	*p*	*B* (95%CI)	*p*
Age	0.01 (–0.10 to 0.12)	.854		
Occupation				
Own business and others	0.00		0.00	
Employed	−1.72 (–2.75 to 0.68)	.001	−1.46 (–2.51 to −0.40)	.007
Ability to pay for medication				
Very or fairly difficult	0.00			
Very or fairly easy	0.17 (–0.74 to 1.09)	.712		
S-COVID-19-S				
No	0.00			
Yes	0.43 (–0.69 to 1.56)	.450		
Comorbidity				
None	0.00			
One or more	−1.31 (–3.57 to 0.95)	.257		
Gestational age				
1st trimester	0.00		0.00	
2nd trimester	0.29 (–0.89 to 1.47)	.624	0.43 (–0.74 to 1.59)	.474
3rd trimester	1.02 (–0.04 to 2.08)	.060	0.73 (–0.32 to 1.79)	.173
Gestational weight gain	0.04 (–0.05 to 0.12)	.392		
Parity				
1	0.00			
≥2	0.85 (–0.06 to 1.77)	.068	0.81 (–0.10 to 1.71)	.081
Eating behaviour				
Unchanged or less-healthy diet	0.00			
Healthier diet	0.33 (–0.58 to 1.24)	.478		
Smoking				
No	0.00			
Yes	0.72 (–1.09 to 2.54)	.435		
Drinking alcohol				
No	0.00			
Yes	0.052 (–1.16 to 1.26)	.933		
Physical activity				
Never, stopped or exercise less	0.00			
Unchanged or exercise more	−1.05 (–1.96 to −0.14)	.023	−0.741(–1.66 to 0.18)	.116
HL index	−0.003 (–0.05 to o.05)	.919		
HES	−0.09 (–0.18 to 0.003)	.058	−0.067 (–0.160 to 0.026)	.160

S-COVID-19-S: suspected COVID-19 symptoms; HL index: health literacy index; HES: healthy eating score; FCoV-19S: fear of COVID-19 scale.

### Associated factors of anxiety

In the bivariate models (Table 3), S-COVID-19-S, gestational age, eating behaviour, smoking, drinking, physical activity, HL index and HES were associated with anxiety at *p* < .20. A moderate correlation between smoking and drinking was found (*rho* = 0.71) (Supplementary Table 1), and drinking was removed from the multivariate model. As shown in Table 3, elevated gestational age (OR = 4.59, 95%CI, 1.37, 15.32, *p*=.013 for second trimester; OR = 4.56, 95%CI, 1.50, 13.83, *p*=.007 for third trimester), and smoking (OR = 4.29, 95%CI, 1.40, 13.14, *p*=.011) were associated with a higher anxiety likelihood. Whereas, having healthier diet (OR = 0.33, 95%CI, 0.12, 0.88, *p*=.026), unchanged or more physical activity (OR = 0.34, 95%CI, 0.13, 0.89, *p*=.029), higher HL (OR = 0.94, 95%CI, 0.90, 0.99, *p*=.018) and higher HES score (OR = 0.79, 95%CI, 0.73, 0.87, *p* < .001) were associated with a lower anxiety likelihood.

**Table 3.** Associated factors of anxiety in pregnant women.

Variables	Anxiety (GAD ≥8)				
	Bivariable		Multivariable		
	OR (95%CI)	*p*	OR (95%CI)		*p*
Age	1.05 (0.97–1.13)	.236			
Occupation					
Own business and others	1.00				
Employed	0.97 (0.47–1.99)	.930			
Ability to pay for medication					
Very or fairly difficult	1.00				
Very or fairly easy	0.88 (0.47–1.67)	.705			
S-COVID-19-S					
No	1.00		1.00		
Yes	0.50 (0.19–1.31)	.160	0.62 (0.21–1.85)		.396
Comorbidity					
None	1.00				
One or more	1.85 (0.53–6.54)	.337			
Gestational age					
1st trimester	1.00		1.00		
2nd trimester	2.94 (0.99–8.67)	.051	4.59 (1.37–15.32)		.013
3rd trimester	4.19 (1.56–11.29)	.004	4.56 (1.50–13.83)		.007
Gestational weight gain	1.02 (0.97–1.09)	.404			
Parity					
1	1.00				
≥2	1.04 (0.55–1.97)	.904			
Eating behaviour					
Unchanged or less-healthy diet	1.00		1.00		
Healthier diet	0.19 (0.08–0.44)	<.001	0.33 (0.12–0.88)		.026
Smoking					
No	1.00				
Yes	10.86 (4.99–23.60)	<.001	4.29 (1.40–13.14)		.011
Drinking alcohol					
No	1.00				
Yes	2.68 (1.35–5.33)	.005			
Physical activity					
Never, stopped or exercise less	1.00		1.00		
Unchanged or exercise more	0.37 (0.18–0.76)	.006	0.34 (0.13–0.89)		.029
HL index	0.97 (0.93–1.00)	.086	0.94 (0.90–0.99)		.018
HES	0.79 (0.73–0.85)	<.001	0.79 (0.73–0.87)		<.001

S-COVID-19-S: suspected COVID-19 symptoms; HL index: health literacy index; HES: healthy eating score.

### Associated factors of depression

Confounders of depression were similar to those of anxiety in the bivariate models (Table 4). Therefore, S-COVID-19-S, gestational age, eating behaviour, smoking, physical activity, HL index and HES were put in the adjusted model. As shown in Table 4, elevated gestational age (OR = 5.45, 95%CI, 1.88, 15.76, *p*=.002 for second trimester; OR = 3.14, 95%CI, 1.13, 8.70, *p*=.028 for third trimester) and smoking (OR = 4.99, 95%CI, 1.85, 13.46, *p*=.002) were associated with a higher depression likelihood. However, having healthier diet (OR = 0.33, 95%CI, 0.13, 0.82, *p*=.017), higher HL (OR = 0.96, 95%CI, 0.91, 0.99, *p*=.044) and higher HES score (OR = 0.84, 95%CI, 0.78, 0.91, *p* < .001) were associated with a lower depression likelihood.

**Table 4.** Associated factors of depression in pregnant women.

Variables	Depression (PHQ ≥10)			
	Bivariable		Multivariable	
	OR (95%CI)	*p*	OR (95%CI)	*p*
Age	1.02 (0.94–1.09)	.648		
Occupation				
Own business and others	1.00			
Employed	1.66 (0.75–3.65)	.211		
Ability to pay for medication				
Very or fairly difficult	1.00			
Very or fairly easy	1.15 (0.63–2.13)	.648		
S-COVID-19-S				
No	1.00			
Yes	0.83 (0.37–1.84)	.645		
Comorbidity				
None	1.00			
One or more	1.71 (0.49–6.02)	.403		
Gestational age				
1st trimester	1.00		1.00	
2nd trimester	3.43 (1.29–9.09)	.013	5.45 (1.88–15.76)	.002
3rd trimester	3.14 (1.24–7.96)	.016	3.14 (1.13–8.70)	.028
Gestational weight gain, median	1.03 (0.98–1.09)	.246		
Parity				
1	1.00			
≥2	0.88 (0.48–1.62)	.679		
Eating behaviour				
Unchanged or less-healthy diet	1.00		1.00	
Healthier diet	0.21 (0.09–0.45)	<.001	0.33 (0.13–0.82)	.017
Smoking				
No	1.00			
Yes	11.35 (5.28–24.38)	<.001	4.99 (1.85–13.46)	.002
Drinking alcohol				
No	1.00			
Yes	3.53 (1.57–5.86)	.001		
Physical activity				
Never, stopped or exercise less	1.00		1.00	
Unchanged or exercise more	0.53 (0.28–1.02)	.057	0.54 (0.24–1.24)	.149
HL index	0.97 (0.94–1.00)	.126	0.96 (0.91–0.99)	.044
HES	0.82 (0.77–0.88)	<.001	0.84 (0.78–0.91)	<.001

S-COVID-19-S: suspected COVID-19 symptoms; HL index: health literacy index; HES: healthy eating score.

## Discussion

The current study highlights the importance of HES and HL for antenatal mothers’ mental health during the COVID-19 pandemic.

In the present study, women with higher HES scores were less likely to be anxious and depressed. The association between a healthy diet and reduced mental illness was found in previous studies [28,39]. Also, higher nutrient quality was associated with a lower risk of anxiety and depression [40]. In the literature review, diet and nutrition were recommended for the prevention and treatment of depression and anxiety [41,42] due to their role in strengthening the immune system and reducing inflammation and oxide stress [43] to protect against viral infections [44]. However, food security and nutritional problems are being seriously threatened by the COVID-19 crisis, which reduced the accessibility to healthy foods [45]. Hence, HESs and attitudes should be attended as a global priority on preventing health problems caused by the pandemic [46]. The finding implicates that healthy eating should be promoted in pregnant women during the pandemic in order to improve their mental health, and in turn, improve maternal and child’s health outcomes.

We also found that women with higher HL levels had a lower risk of prenatal anxiety and depression. The potential role of HL in protecting people against depression and anxiety during the pandemic was illustrated in previous studies [18,28,47]. Women with higher HL were also better educated and had better access to healthy foods [48]. In addition, HL linked with nutrition practice, which contributed to the pathway of improving health outcomes [49]. This was strengthened by our results that women practicing a healthier diet during the COVID-19 outbreak had a lower likelihood of anxiety and depression than those having an unchanged or less healthy diet. In the context that the HL levels in pregnant women are mixed [50], and the culture of HL has an immense gap during COVID-19 [51], health professionals and policymakers, organizations, communities, families and individuals need to be in a linked, multi-sector effort to improve HL and control mental health problems among pregnant women during the pandemic. The government needs to recognize the COVID-19 outbreak as an emerging public health concern and provides the communities with timely, updated, accurate and brief information and knowledge regarding COVID-19 and mental health matters. Additionally, the individuals should enhance their self-belief, knowledge and health behaviours to develop HL.

In addition, our findings on the association between smoking during pregnancy and the occurrence of both mentioned mental disorders were similar to previous studies. For example, Ceulemans et al. found that higher likelihoods of anxiety and depression were observed in pregnant women who smoked during the COVID-19 pandemic [52]. Likewise, Newport et al. showed that the level of tobacco use throughout gestation was positively associated with the severity of both maternal anxiety and depression [53]. Hence, we assumed that pregnant women with psychological illnesses were more likely to smoke [54,55], and they were less successful in their efforts to quit smoking [56]. Moreover, our results showed that women having an unchanged or higher frequency of physical activity during pregnancy could protect them against anxiety risk, which was similar to the existing literature [57]. Therefore, healthy lifestyles are highly recommended to preventing antenatal psychological ill-being during the COVID-19 pandemic.

Besides, elevated gestational age was found as the identified risk factor of anxiety and depression among pregnant women in the current study. However, reports regarding such association across studies conducted before the COVID-19 pandemic were contradictory. Several studies showed an inverse correlation between gestational age with anxiety [58] and depression [59] in pregnancy. On the contrary, Rezaee and Framarzi shared similar findings that gestational age was positively correlated with anxiety and depression symptoms [60]. Therefore, we supposed that the most prevalent occurrence of pregnancy complications, such as preeclampsia and gestational diabetes mellitus, was in the late pregnancy, which contributed to the decrease of the immune system. Moreover, the reduction of the innate immune responses in pregnant women increased maternal susceptibility to COVID-19 infection that involved high maternal mortality [61,62], which may lead to maternal anxiety and depression symptoms. In addition, pregnant women faced many difficulties during the COVID-19 pandemic, such as limitation in assessing healthcare services [5] and the chance of infection during delivery and hospital visits [63], especially the high risk of delivery in the late pregnancy, which may increase the possibility of anxiety and depression.

Lastly, our results revealed that employed pregnant women had significantly lower fear of COVID-19 scores than those with other types of occupation. This was similar to a previous finding reported by Jafree et al. that unemployed women had a greater fear of contracting COVID-19 than those employed [64]. Similarly, Matsushima and Horiguchi indicated that losing employment was positively associated with the mental ill-being of pregnant women [65]. Several reasons could explain this association. First, unemployed people face a high risk of infectious diseases [66], which may increase the fear of virus exposure. Second, employees' psychological safety is improved positively by inclusive leadership at the workplace [67], resulting in lower fear emotion of employed labours.

Thus far, the current study is the first to assess the factors associated with mental health problems among pregnant women in Vietnam. The current findings contribute the initial evidence for further research and future preventive programs related to maternal mental health. However, several limitations should be noted in this study. First, participants and interviewers were vulnerable to coronavirus exposure as the study was conducted in the global outbreak of COVID-19, which requires their great effort on strictly following the safety guidelines during data collection. Second, in a cross-sectional study, causality cannot be implied, only associations were recognized. Lastly, several indicating factors of mental health were not assessed in our studies, such as the history of depression before pregnancy, maternal education and income, but we assessed the ability to pay for medication instead.

## Conclusions

Among investigated factors, HES and better HL had critical roles in lowering prenatal anxiety and depression during the COVID-19 crisis. Therefore, strategic approaches for improving healthy eating and HL should be recommended for protecting pregnant women from mental health problems during the pandemic.

## Supplementary Material

Supplemental Material

## Data Availability

The raw data supporting the conclusions of this article will be made available on reasonable request to the corresponding author.
