# Supplementary material for: Fear, anxiety and depression among pregnant women during COVID-19 pandemic: impacts of healthy eating behaviour and health literacy
Source: Ann Med. 2021 Nov 11;53(1):2120–31. doi: 10.1080/07853890.2021.2001044 (PMC8592601; doi:10.1080/07853890.2021.2001044)
Supplement: Supplemental Material [file IANN_A_2001044_SM4211.docx]

Supplementary Table 1: Spearman’s correlations (rho) among the studied variables (n= 518).

| Variables | Occupation | S-COVID-19-S | Gestational age | Parity | Eating behavior | Smoking | Drinking alcohol | Physical activity | HL index |
| --- | --- | --- | --- | --- | --- | --- | --- | --- | --- |
| S-COVID-19-S | 0.11 |  |  |  |  |  |  |  |  |
| Gestational age | 0.10 | -0.03 |  |  |  |  |  |  |  |
| Parity | 0.00 | 0.05 | 0.06 |  |  |  |  |  |  |
| Eating behavior | -0.15 | -0.08 | 0.03 | -0.04 |  |  |  |  |  |
| Smoking | 0.03 | -0.07 | 0.06 | -0.02 | -0.07 |  |  |  |  |
| Drinking alcohol | 0.04 | -0.05 | 0.07 | -0.03 | -0.10 | 0.71 |  |  |  |
| Physical activity | -0.20 | -0.09 | -0.07 | -0.05 | 0.17 | 0.11 | 0.12 |  |  |
| HL index | -0.15 | -0.23 | 0.01 | -0.10 | 0.22 | 0.08 | 0.12 | 0.23 |  |
| HES | -0.08 | 0.03 | -0.02 | 0.01 | 0.18 | -0.14 | -0.13 | 0.11 | 0.06 |

Abbreviations: *S-COVID-19-S, suspected COVID-19 symptoms; HL index, health literacy index; HES, healthy eating score.*
